# From No Disease to Stage IV Colon Cancer in Four Months: A Case Report

**DOI:** 10.7759/cureus.58134

**Published:** 2024-04-12

**Authors:** Antoine Jeri-Yabar, Liliana Vittini-Hernandez, Renzo Aller-Rojas, Francisco Arias-Reyes, Claudia Lozada Zingoni

**Affiliations:** 1 Department of Internal Medicine, Icahn School of Medicine at Mount Sinai Beth Israel, New York, USA; 2 Department of Medicine, Icahn School of Medicine at Mount Sinai Beth Israel, New York, USA; 3 Department of Internal Medicine, Clinica Anglo Americana, Lima, PER; 4 Department of Medical Oncology, Clinica Anglo Americana, Lima, PER

**Keywords:** stage iv colon cancer, screening, oncology, steroid, colorectal cancer

## Abstract

Colorectal cancer remains one of the most common cancers in the population. Meanwhile, steroids or other immunosuppressive drugs are usually given in rheumatological diseases as a treatment for flare-ups. Herein, we present the case of a 61-year-old female diagnosed with metastatic colorectal cancer merely four months following the commencement of glucocorticoid therapy for a recently diagnosed rheumatologic condition, despite a clear colorectal cancer screening colonoscopy conducted four months prior. The case report discusses the possible impact of corticosteroids on the fast disease progression of colorectal cancer and raises awareness regarding this potential risk.

## Introduction

Colon cancer is the most common gastrointestinal cancer and the second leading cause of cancer that leads to death worldwide. Incidence has increased in the past years in people younger than 65 years of age with a 1% increase annually and 2% in people younger than 50 years in 2020 [1]. 

The immune system plays a critical role in eliminating pathogens and eradicating tumorigenic cells in the colon; it has been shown that when this is altered, there is a higher risk of autoimmune diseases and colon cancer [2]. Corticosteroid therapy as an immunosuppressant has been shown to be a key factor in the disease progression of colon cancer since the immune system plays an essential role in preventing colorectal malignancy [3].

Herein, we present the case of a 61-year-old woman with transverse colon tubular adenocarcinoma diagnosed at stage IV, due to sudden onset and rapid progression within only four months of corticosteroid treatment for her recently diagnosed spondyloarthritis. Although we cannot conclude that immunosuppressant therapy accelerated the sudden diagnosis, it is essential to construct this case presentation to spark a conversation about screening for malignancies in a frequent pattern before, during, and after immunosuppressive therapy such as corticosteroids.

## Case presentation

A 61-year-old non-smoker woman presented to our Emergency Department with complaints of three days of right upper quadrant (RUQ) pain and nausea. The pain was described as dull and constant, with no alleviating or exacerbating factors reported. The onset of symptoms was gradual over the course of three days, progressively worsening in intensity.

On physical examination, the patient's vital signs were within normal limits, with a blood pressure of 120/80 mmHg, heart rate of 80 beats per minute, respiratory rate of 16 breaths per minute, and temperature of 37°C. Abdominal examination revealed tenderness on superficial palpation in the RUQ, without rebound tenderness or guarding. Bowel sounds were present in all quadrants, and there were no signs of jaundice or palpable masses. Examination of other systems, including cardiovascular, respiratory, and neurological, was unremarkable.

**Figure 1:**
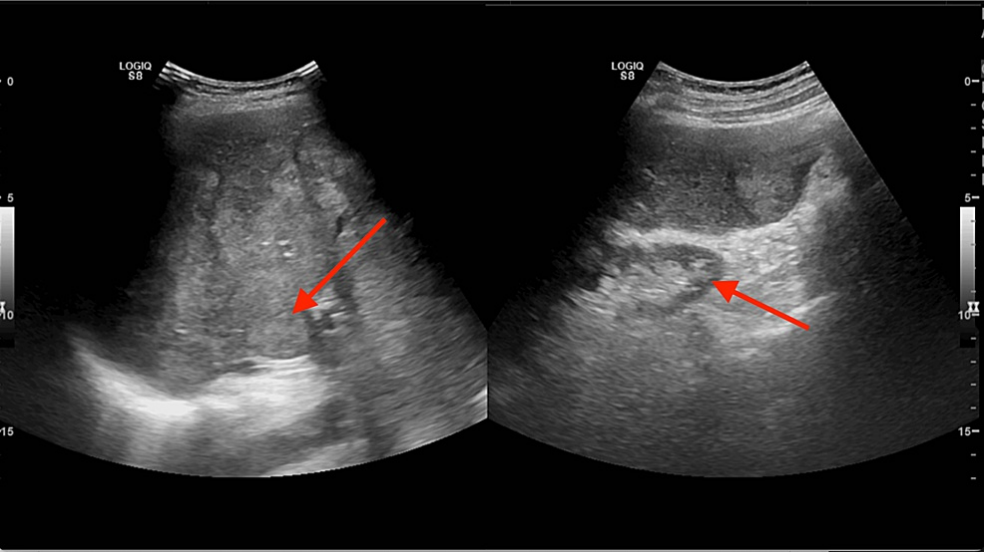
Abdominal ultrasound showing nodular lesions in the liver (red arrows)

Given the clinical presentation suggestive of possible biliary pathology, an ultrasound of the abdomen was promptly ordered. The ultrasound revealed multiple nodular lesions scattered throughout the liver parenchyma, ranging in size from a few millimeters to the largest lesion measuring 8 cm in diameter (Figure 1). The appearance of these lesions raised concerns for metastatic disease, prompting further investigation.

**Figure 2:**
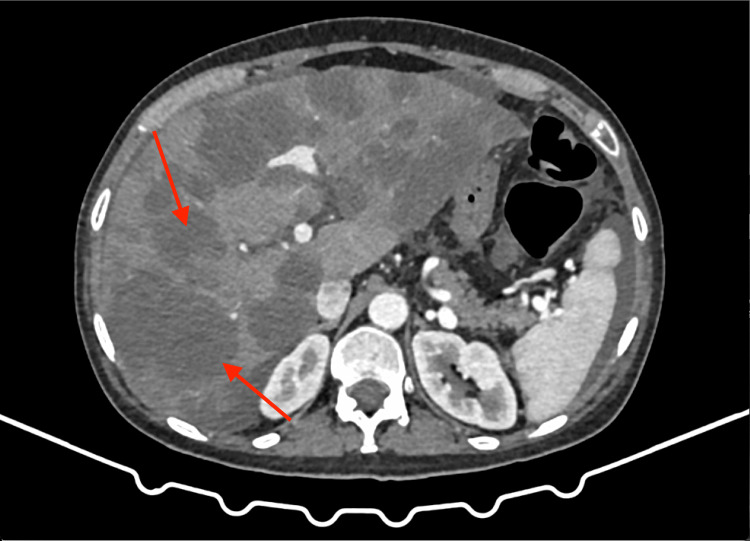
CT of the abdomen showing multiple liver masses concerning for metastases (red arrows)

**Figure 3:**
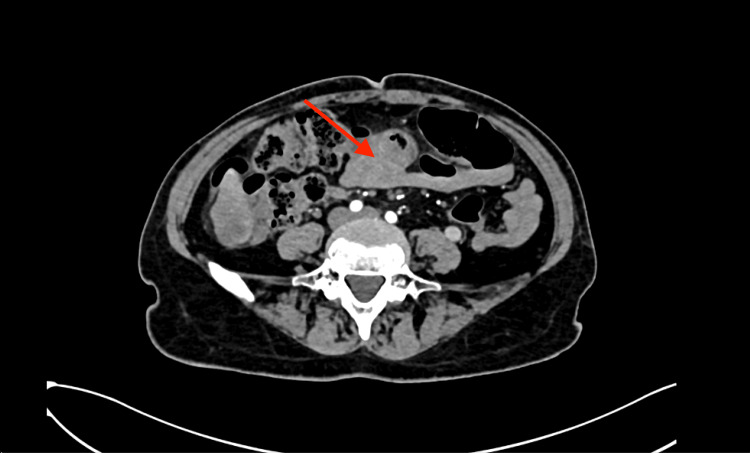
CT of the abdomen showing transversal colon mass (red arrows)

Subsequent imaging with abdominal CT was performed to further evaluate the liver lesions and assess for additional intra-abdominal pathology. The CT scan confirmed the presence of multiple hepatic lesions, with enhancement patterns consistent with metastatic deposits. In addition, parietal thickening of the transverse colon was noted, along with adjacent mesenteric adenopathy and evidence of peritoneal carcinomatosis. These findings raised suspicion for primary gastrointestinal malignancy with metastatic spread to the liver and surrounding structures (Figure 2 and Figure 3).

**Figure 4:**
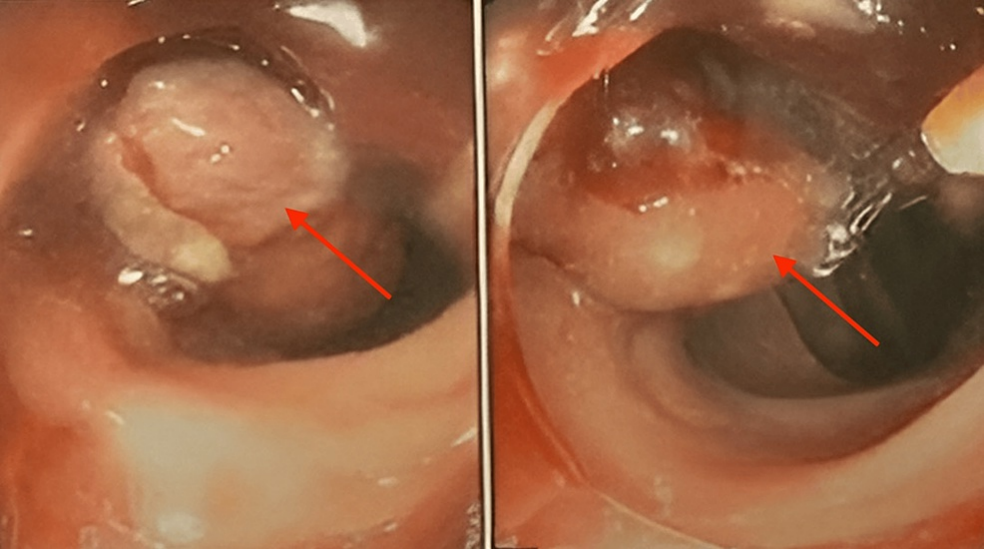
Colonoscopy showing colonic mass with more than 50% obstruction

Given the suspicion of a primary gastrointestinal malignancy, a colonoscopy was scheduled to further investigate the colonic lesion. Colonoscopy revealed a large, friable mass obstructing approximately 50% of the lumen in the transverse colon (Figure 4). Biopsy samples were obtained from the lesion, and histopathological examination confirmed the presence of poorly differentiated adenocarcinoma, consistent with metastatic spread from the colon. 

**Figure 5:**
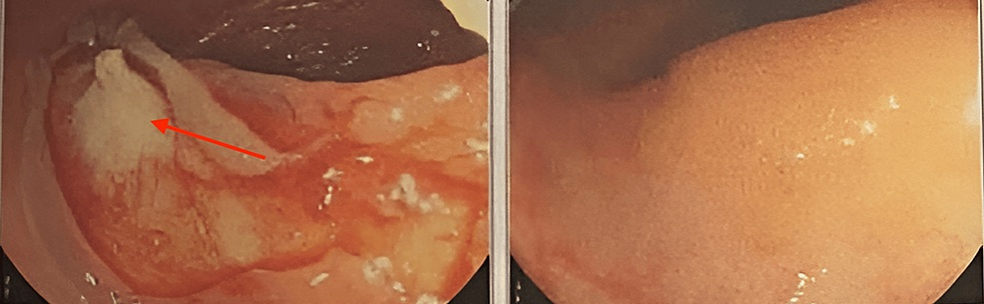
Prior colonoscopy showing sessile polyp (red arrow), otherwise no lesions

The patient's medical history was significant for a recent diagnosis of spondyloarthritis in August 2022, for which she had been prescribed methylprednisolone (15 mg PO daily) by her rheumatologist. Prior to initiating treatment for spondyloarthritis, the patient underwent screening tests, including abdominal ultrasound and colonoscopy, as part of routine surveillance due to her age and medical history. A 12 mm polyp was identified near the hepatic angle during colonoscopy, which was completely excised by mucosectomy and confirmed to be a benign sessile tubular adenoma of the colon (Figure 5). Additionally, the patient had a history of rectal squamous cell carcinoma diagnosed in 2010, which had been successfully treated with chemotherapy and radiation therapy. Follow-up imaging studies, including positron emission tomography (PET) scans, had shown no evidence of disease recurrence, with the last scan performed six months prior to the current presentation.

Upon confirmation of the diagnosis of metastatic colon adenocarcinoma, the patient was referred to the oncology team for further management. A multidisciplinary approach was adopted, and the patient was initiated on FOLFOX (folinic acid, fluorouracil, and oxaliplatin) chemotherapy regimen combined with bevacizumab, a monoclonal antibody targeting vascular endothelial growth factor (VEGF). The patient tolerated the treatment well initially, with no significant adverse effects reported during the initial cycles of chemotherapy; however, she had a progression of disease and unfortunately passed away recently. 

## Discussion

Colorectal cancer (CRC) is the third most common cancer with an estimated number of 1.8 million new cases per year with the recent tendency of increasing incidence in the younger population shown in a report from 2021 [1]. However, it is well known that the epidemiology of CRC varies among regions, genders, and racial groups; this is because of multiple underlying factors such as risk factor exposure, genetic susceptibility of each patient, and lifestyle [4]. 

The development of CRC begins primarily with polyps which are found on the lumen of the colon, and the risk of malignancy varies depending on the histopathological features of the polyp. Villous adenomas are the riskier ones with a 40% chance of becoming CRC [5]. In the patient presented above, the colonoscopy done four months prior to diagnosis did reveal a polyp, but it was a sessile tubular polyp which only has a 5% risk of becoming malignant in the next 10-15 years. Patients with stage IV CRC are usually treated with fluorouracil-based chemotherapies and VEFG inhibitors or epidermal growth factor receptor (EGFR) inhibitors. Furthermore, the life expectancy is discouraging with it only being in the range of 21.3-30.1 months [2]. 

It is important to note that the development of our patient's cancer was high speed since she had a clean successful colonoscopy four months prior to her CRC stage IV diagnosis; the only change in lifestyle and medication was the addition of methylprednisolone because of a recent diagnosis of spondyloarthritis. To our knowledge, only one other case report has been published showing rapid progression of colonic mucinous adenocarcinoma within four months of immunosuppressive therapy with methylprednisolone in a young female [6]. This sparks the conversation about the possible link between CRC appearance and immunosuppressive therapy. The dynamics of tumor initiation remain undetermined since multiple factors play a role, but immunosuppressive therapy has been associated with increased incidence of various types of cancer and glucocorticoids with tumor progression [2]. 

The use of corticosteroids has been associated with an increased risk of colon cancer, likely due to their immunosuppressive effects [7]. In contrast, Ostenfeld et al. performed a case-control study in Northern Denmark where they found that frequent use of systemic glucocorticoids (defined as >2 prescriptions) was not associated with the risk of CRC [8]. However, Lai et al. responded to this by showing that in their cohort study conducted in the Taiwanese population, oral use, injection use, and the topical use of glucocorticoids and the risk of colon cancer were in fact associated since the case group had a higher incidence of CRC as per the control group (IR 1.16 vs 0.81 per 1000 person-years, 95% CI=1.30, 1.58) [9]. This conflicting result of two big studies in different populations with the addition of the case report presented should spark further investigation on this matter to confirm the present role of glucocorticoid use on the risk of CRC as well as the speed of progression. 

Regular colon cancer surveillance, often conducted through colonoscopy, plays a pivotal role in identifying early abnormalities and significantly enhancing the prospects of successful management [10]. Despite the existence of multiple guidelines delineating surveillance protocols for colon cancer in individuals classified as low, moderate, or high risk, the optimal surveillance regimen remains uncertain for patients undergoing corticosteroid therapy. Hence, it is imperative for individuals on corticosteroids to engage in comprehensive discussions with their physicians regarding the most suitable surveillance schedule tailored to their unique risk profiles. Further research is essential to ascertain the most effective surveillance strategy for this particular patient cohort.

## Conclusions

We present a case study detailing a 61-year-old woman diagnosed with stage IV transverse CRC, which manifested within four months and exhibited rapid progression subsequent to the commencement of corticosteroid therapy for rheumatological comorbidity. While further cohort studies are warranted to substantiate the correlation between immunosuppressive corticosteroid therapy and heightened CRC incidence, it is imperative to establish guidelines for conducting colonoscopy and endoscopy surveillance screenings before, during, and after immunosuppressive treatment in order to facilitate early detection of the disease.
